# Adult spinal deformity and its relationship with height loss: a 34-year longitudinal cohort study

**DOI:** 10.1186/s12891-020-03464-2

**Published:** 2020-07-01

**Authors:** Mutsuya Shimizu, Tetsuya Kobayashi, Hisashi Chiba, Issei Senoo, Hiroshi Ito, Keisuke Matsukura, Senri Saito

**Affiliations:** 1 Department of Orthopaedic Surgery, Asahikawa Medical University, 2-1E Midorigaoka, Asahikawa, Hokkaido 078-8510 Japan; 2 Department of Rehabilitation and Physical Therapy, Furano Kyokai Hospital, Furano, Japan; 3 Department of Orthopaedic Surgery, Furano Kyokai Hospital, Furano, Japan

**Keywords:** Adult spinal deformity, Height loss, Lumbar lordosis, Spondylosis, Lumbar scoliosis, Thoracic kyphosis, Pelvic tilt, Sagittal vertical axis

## Abstract

**Background:**

Age-related height loss is a normal physical change that occurs in all individuals over 50 years of age. Although many epidemiological studies on height loss have been conducted worldwide, none have been long-term longitudinal epidemiological studies spanning over 30 years. This study was designed to investigate changes in adult spinal deformity and examine the relationship between adult spinal deformity and height loss.

**Methods:**

Fifty-three local healthy subjects (32 men, 21 women) from Furano, Hokkaido, Japan, volunteered for this longitudinal cohort study. Their heights were measured in 1983 and again in 2017. Spino-pelvic parameters were compared between measurements obtained in 1983 and 2017. Individuals with height loss were then divided into two groups, those with degenerative spondylosis and those with degenerative lumbar scoliosis, and different characteristics were compared between the two groups.

**Results:**

The mean age of the subjects was 44.4 (31–55) years at baseline and 78.6 (65–89) years at the final follow-up. The mean height was 157.4 cm at baseline and 153.6 cm at the final follow-up, with a mean height loss of 3.8 cm over 34.2 years. All parameters except for thoracic kyphosis were significantly different between measurements taken in 1983 and 2017 (*p* < 0.05). Height loss in both sexes was related to changes in pelvic parameters including pelvic incidence-lumbar lordosis (*R =* 0.460 *p =* 0.008 in men, *R =* 0.553 *p =* 0.012 in women), pelvic tilt (*R =* 0.374 *p =* 0.035 in men, *R =* 0.540 *p =* 0.014 in women), and sagittal vertical axis (*R =* 0.535 *p =* 0.002 in men, *R =* 0.527 *p* = 0.017 in women). Greater height loss was more commonly seen in women (*p =* 0.001) and in patients with degenerative lumbar scoliosis (*p =* 0.02).

**Conclusions:**

This longitudinal study revealed that height loss is more commonly observed in women and is associated with adult spinal deformity and degenerative lumbar scoliosis. Height loss is a normal physical change with aging, but excessive height loss is due to spinal kyphosis and scoliosis leading to spinal malalignment. Our findings suggest that height loss might be an early physical symptom for spinal malalignment.

## Background

Population aging is seen worldwide. Thus, it is important that an appropriate quality of life (QOL) is maintained for the entire lifespan of any individual. Adult spinal deformity (ASD) can affect the sagittal alignment of the spine, which may impact the QOL [1–3]. The ultimate aim of ASD treatment, even in younger patients, is to ensure that patients are ambulant without requiring a cane or handrail for support. ASD affects muscular strength leading to changes in the range of motion of the trunk, lower extremities, and the whole body [4, 5].

Individuals with ASD will receive lower scores in all domains of the 36-Item Short Form Survey as they age, while in other chronic conditions such a decline with age is not observed [6]. For evaluating ASD, ideal values for parameters including pelvic incidence-lumbar lordosis (PI-LL), pelvic tilt (PT), and sagittal vertical axis (SVA) were proposed by the International Spine Study Group [3], and target values have been modified to include age and surgical results [7–9].

Height loss is an age-related physical change that occurs in all individuals over 50 years and that elderly people are aware of [10], and is correlated with osteoporosis [11, 12], vertebral fracture [13–16], and mortality [17]. Decreased QOL is also reportedly associated with height loss according to the EQ-5D scores [18].

Although both ASD and height loss are associated with aging, no study has examined the relationship between height loss and ASD, based on the abovementioned parameters. Besides, many epidemiological studies were conducted on height loss worldwide; however, over the 30 years longitudinal no epidemiological studies have been conducted in this field. The purpose of this 34-year longitudinal cohort study was to investigate the relationship between ASD and height loss in community-based volunteers and to determine the effects of sex.

## Methods

### Cohort selection

This study is a follow-up of an epidemiological study by Takemitsu et al. in 1983, in Furano, Hokkaido, Japan [19]. Of the 249 adult volunteers who lived locally, and who had participated in the original study in 1983, we included 53 volunteers (32 men/21 women) in 2017 for this follow-up 34-year longitudinal study. The subjects were living in an agriculture area and trunk bending positions were a requirement of their job. The initial recruitment of the cohort was done by sending invitations to all rural residents of the area using the information in a resident ledger. In the current study, those who had participated in the initial study were contacted by phone and invited to take part in this survey.

The inclusion criteria were as follows: no spinal or major joint surgery between 1983 and 2017, ability to walk independently to our hospital for the present study, and aged less than 55 years in 1983 in consideration of current age.

**Fig. 1:**
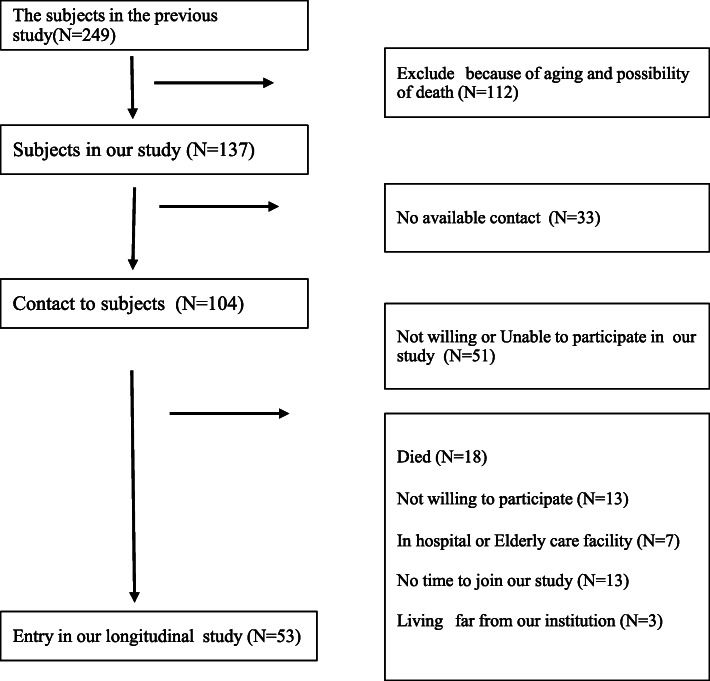
Flowchart of the enrolment process in our study. We approached 104 local subjects, of which 53 agreed to participate in this study

The exclusion criteria were being unable to stand in the correct posture during radiography, unable to tolerate the pain, osteoporosis fracture, paralysis of trunk or lower extremity, and not consenting to the study. Figure 1 presents the subject details.

### Spinal X-ray

The subjects underwent radiography of the entire spine in an upright position in 1983 and 2017. The participants were instructed to stand in a relaxed position and look straight ahead, with their fists at the level of the clavicle.

The heights of all subjects were measured in meters without their shoes, with the back of their head, buttocks, back, and heels against an upright board. The back muscle was stretched as much as possible, and feet were supinated by about 30°. Participants with height loss were then selected and divided into two groups: those with ≤4 cm (small height less, S group) and those with > 4 cm (large height loss, L group) loss of height.

### Radiographic parameters

**Fig. 2:**
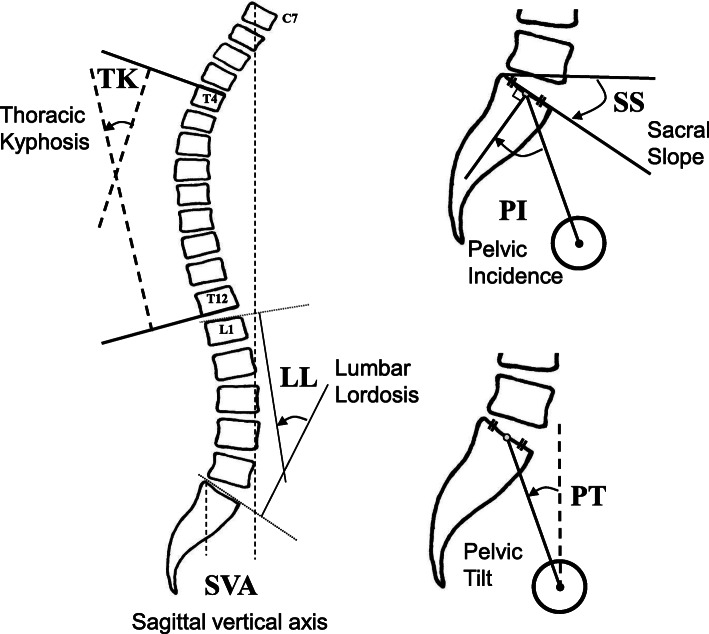
Radiographic measurements of spinopelvic alignment. Left: thoracic kyphosis (TK), lumbar lordosis (LL), sagittal vertical axis (SVA). Right: pelvic incidence (PI), sacral slope (SS), and pelvic tilt (PT)

Standardized radiographic measurements of sagittal spino-pelvic parameters included thoracic kyphosis (TK), lumbar lordosis (LL), PT, pelvic incidence (PI), sacral slope (SS), and SVA [20, 21] (Fig. 2). ASD was radiologically evaluated based on the SRS-Schwab ASD classification sagittal modifiers [3]. Degenerative spondylosis (DS) was determined as 5% percent slip [22] and degenerative lumbar scoliosis (DLS) was identified based on the Cobb method [23] with a scoliotic angle of more than 10° [24–26].

### Statistical analysis

All statistical analyses were performed using SPSS Statistics version 12 (IBM, Tokyo, Japan). All data are expressed as mean and range. Variables that were normally distributed were analyzed using the Shapiro-Wilk test. When measured variables were found to have a non-normal distribution, Spearman’s correlation was used to evaluate the association between height loss and spino-pelvic parameters. Sub-analyses were performed to evaluate the association between height loss and sex, DS, and DLS.

In comparing the height loss between the 2 groups, if lack of normality was rejected, a t-test was performed. If the lack of normality was significant, the Wilcoxon signed rank test or Mann-Whitney U-test was performed, as appropriate. A *P-*value of < 0.05 was considered statistically significant.

## Results

The mean age of the subjects was 44.4 (31–55) years at baseline and 78.6(65–89) years at the final follow-up. The mean follow-up period was 34.2 (34–34.4) years. The mean height of the subjects was 157.4 (140–170) cm at baseline and 153.6(132–169) cm at the final follow-up, with a mean height loss of 3.8 cm over 34 years.

A summary of longitudinal changes in radiographic variables from baseline to the final follow-up is presented in Table 1. Statistical analyses revealed significant longitudinal changes in all parameters except for body mass index and TK.

**Table 1:** Changes in characteristics of participants after 34 years^1^

Variables	Baseline	Final follow-up	***P*** value^**2**^
Age (years)	44.4 (31 to 55)	78.6 (65 to 89)	< 0.0001
Height (cm)	157.4 (140 to 170)	153.6 (132 to 169)	< 0.0001
Body weight (kg)	61.9 (44 to 85)	58.0 (38 to 87)	< 0.0001
BMI (kg/m^2^)	24.9 (20 to 31)	24.4 (18 to 30)	0.203
TK	35.4 (16 to 66)	34.3 (2 to 69)	0.515
LL	55.1 (32 to 79)	32.4 (−32 to 77)	< 0.0001
PT	14.4 (0 to 39)	25.9 (4 to 54)	< 0.0001
PI	56.0 (33 to 76)	54.4 (27 to 78)	0.015
PI-LL	0.9 (− 25 to 30)	21.1 (−15 to 84)	< 0.0001
SS	41.4 (26 to 58)	28.7 (0 to 48)	< 0.0001
SVA	4.3 (−40 to 67)	63.9 (−31 to 282)	< 0.0001

^1^Data are presented as mean (range)

^2^*P* value < 0.05 was considered significant

Abbreviations: *BMI* body mass index, *TK* thoracic kyphosis, *LL* lumbar lordosis, *PT* pelvic tilt, *PI* pelvic incidence, *SS* sacral slope, *SVA* sagittal vertical axis

Spino-pelvic parameters including PI-LL, PT, and SVA were significantly associated with height loss in both male and female subjects (Table 2). Based on our definition for DS, 7 participants were in the DS(+) group and 46 participants in the DS(−) group. Upon assessing for DLS, 26 participants were in the DLS(+) group and 27 in the DLS(−) group. The large height loss group had more patients with DLS, but there were no significant differences in height loss between the DS groups (Tables 3, 4). When we compared height loss in terms of sex, 31% of the participants in the large height loss group were men and 76% were women, suggesting that female subjects had greater height loss than male subjects (Table 5).

**Table 2:** Spearman’s correlations among different parameters in male and female participants

Variables	Male		Female	
	Correlation coefficient	***P*** value^1^	Correlation coefficient	***P*** value^1^
Weight	0.438	0.012	0.187	0.427
BMI	0.302	0.093	0.136	0.567
TK	−0.058	0.752	−0.244	0.300
LL	0.438	0.014	0.530	0.016
PT	0.374	0.035	0.540	0.014
PI	0.165	0.366	0.06	0.801
PI-LL	0.460	0.008	0.553	0.012
SS	−0.432	0.014	−0.635	0.003
SVA	0.535	0.002	0.527	0.017

^1^*P* value < 0.05 was considered significant

Abbreviations: *BMI* body mass index, *TK* thoracic kyphosis, *LL* lumbar lordosis, *PT* pelvic tilt, *PI* pelvic incidence *SS* sacral slope, *SVA* sagittal vertical axis

**Table 3:** Comparison between degenerative spondylosis and height loss

	L group (> 4 cm) [***N*** = 26]	S group (≤4 cm) [***N*** = 27]	Total
DS (+)	2	5	7
DS (−)	24	22	46
Total	26	27	53

*P-*value = 0.226 using a χ^2^ -test.

*DS* degenerative spondylosis.

**Table 4:** Comparison between degenerative lumbar scoliosis and height loss

	L group (> 4 cm) [***N*** = 26]	S group (≤4 cm) [***N*** = 27]	Total
DLS (+)	17	9	26
DLS (−)	9	18	27
Total	26	27	53

*P-*value = 0.02 using a χ^2^ -test.

*DLS* degenerative lumbar scoliosis.

**Table 5:** Comparison of height loss between the sexes

	L group (> 4 cm) [***N*** = 26]	S group (≤4 cm) [***N*** = 27]	Total
Male (%)	10 (31%)	22 (69%)	32
Female (%)	16 (76%)	5 (24%)	21
Total	26	27	53

*P* value =0.001 using a χ^2^ -test.

## Discussion

To the best of our knowledge, this 34-year longitudinal cohort study is the first to examine the relationship between ASD and height loss. We observed that height loss was significantly correlated with changes in the sagittal modifiers of the SRS-Schwab classification [3]. Our results showed that height loss was more common in female subjects and was related to coronal and sagittal deformities observed in DLS and ASD.

There was no significant change in the values for TK over 34 years, which is similar to the findings of Kobayashi et al. who also reported small changes in TK over their study period of 12.3 years [4]. On the other hand, Diebo et al. reported a wider range for TK, with spinal malalignment ranging from hyper- to hypokyphosis [27], which might have been because of the wide range of spine flexibility, disc degenerations, and age observed in their cohort. Kamimura also showed that spinal kyphosis was significantly associated with height loss in elderly Japanese women [28]. Their report used self-reported categories of kyphosis, which though simple, was not objective.

Aging-related spinal changes begin with degenerative inter-vertebral disk changes [29]. Later, height loss may occur because of several causes, such as progression of intervertebral disk degeneration, vertebral deformity or fracture, and decreased muscle strength, all of which are found in subjects with ASD. Hence, radiographic parameters are useful tools for subjective evaluation of ASD, since changes in SVA, PT, and PI-LL might also lead to height loss.

The main cause for DLS is disc degeneration, leading to decreased disc height and the progression of DLS. Faraj et al. provided strong evidence that increased disc degeneration in DLS, the first sign of height loss, leads to progression of the lumbar scoliosis curve [30]. In the current study, more subjects were diagnosed with DLS compared to the general population. Such a high incidence might be due to the fact that most people living in this area are agricultural workers in the area, and the inherent requirement of the job is that they should remain in a forward bending position for long periods of time. This position increases the load on the lumbar spine, which can be a cause for the higher prevalence of degenerative scoliosis in this cohort.

Furthermore, it is reported that LL decreases and SVA increases as degenerative scoliosis progresses [31], with preceding coronal and sagittal deformities also influencing each other [29]. Hence, scoliosis is reportedly one of the factors associated with height loss of 3 cm or more [11].

In our study, height loss was more pronounced in women, which might be because of the differences in the skeletal structure between the sexes. It should be noted that these differences may be observed over time; however, to date, no prospective studies have confirmed this change, although Takemitsu et al. have reported a higher prevalence of lumbar degenerative kyphosis in women in a previous study [19], which might explain the higher prevalence of height loss in women than in men. Besides, women are reportedly more likely to experience degenerative disk changes [32]. Furthermore, while vertebral deformity is expected to occur in elderly subjects [32], women were at a higher risk of vertebral fractures associated with aging. Reduced muscular strength due to advanced age and vertebral fractures because of osteoporosis mainly occur in women, and the female sex is a risk factor for DLS [33]. Therefore, ASD and height loss are more commonly seen in women. In a 10-year prospective study, Yoshimura reported that in men, height loss did not differ significantly between those in the 40–49 years age group and the 70–79 years age group, but in women, height loss was more common in the 70–79 years age group than in other age groups (40–49 and 60–69 years) [34]. These results suggest that height loss progresses rapidly with age in women.

The complication rate of ASD surgery is high [35, 36], and early care for maintaining sagittal alignment is one of the most important treatments. The use of bisphosphonate and denosumab as an early treatment to maintain spinal alignment and to prevent height loss [37, 38], especially when combined with the reportedly effective physical treatment for spinal deformity, may result in delaying surgical treatment [39, 40].

One of the strengths of our study is that height was measured accurately in both times, while in previous reports, participants recalled their previous heights from memory, which might have led to inaccurate height recording [11]. Our study also had several limitations. Firstly, the number of subjects was low, which affected the findings of our study. However, follow-up periods were longer than previously reported studies. Secondly, our study did not examine QOL score, or living status in relation to height loss. Thirdly, subjects were living in an agriculture area and trunk bending positions were a requirement of their job. Therefore, we cannot generalize our results to other populations. Large longitudinal studies including subjects from more diverse backgrounds are required to address these limitations.

## Conclusions

We observed that height loss was related to ASD and DLS. Height loss was more prominent in women than in men. Height loss is a normal physical change with aging, but excessive height loss is mainly due to the occurrence of spinal kyphosis and scoliosis leading to spinal malalignment. Hence, height loss may be considered as a physical symptom for spinal malalignment.

## Data Availability

The datasets used for the current study are available from the corresponding author upon reasonable request.

## Abbreviations

QOL: Quality of life
ASD: Adult spinal deformity
PI-LL: Pelvic incidence-lumbar lordosis
TK: Thoracic kyphosis
LL: Lumbar lordosis
PT: Pelvic tilt
PI: Pelvic incidence
SS: Sacral slope
SVA: Sagittal vertical axis
DS: Degenerative spondylosis
DLS: Degenerative lumbar scoliosis
